# Melanopsin gene polymorphism and volumetric change in the left hippocampal dentate gyrus following light therapy in mood disorders: An exploratory study

**DOI:** 10.3389/fpsyt.2026.1876421

**Published:** 2026-08-13

**Authors:** Hirofumi Hirakawa, Masaaki Muronaga, Toshihiko Izumi, Kentaro Kohno

**Affiliations:** Department of Neuropsychiatry, Oita University Faculty of Medicine, Oita, Japan

**Keywords:** brain imaging, bright light exposure, hippocampal dentate gyrus, melanopsin, single-nucleotide polymorphisms

## Abstract

**Introduction:**

Bright light therapy (BLT) is an effective treatment for mood disorders. We previously reported that four weeks of bright light (BL) exposure in patients with mood disorders led to increased volume of the left dentate gyrus (DG). We propose a novel hypothesis that the extent of light-induced volumetric increase in the left DG may be influenced by individual genetic variation in the OPN4 gene, especially rs1079610. This exploratory study aimed to test this hypothesis.

**Methods:**

We recontacted 21 patients with mood disorders who had previously participated in a 4-week, randomized controlled trial comparing BL (10,000 lux) and dim light (DL; 50 lux). All participants underwent magnetic resonance imaging and ^18^F-fluorodeoxyglucose (FDG) positron emission tomography at baseline and after 4 weeks of light exposure. Blood samples were collected for genotyping of two Single-Nucleotide Polymorphisms (SNPs) in OPN4 (rs1079610 and rs2675703), which are expressed in intrinsically photosensitive retinal ganglion cells. The primary outcome was the main effect of the rs1079610 genotype for percentage change volume (%ΔV) of the left DG. Analysis of covariance was conducted using a general linear model.

**Results:**

The final sample size was 21 (BL group, n = 11; DL group, n = 10). A significant main effect of rs1079610 genotype (C allele carriers versus TT) was observed (F (1,17) = 7.16, P = 0.016, partial η^2^ = 0.3). Sensitivity analyses were performed with sequential adjustment for potential confounders. The association between rs1079610 genotype and %ΔV of the left DG remained statistically significant after adjustment for age, sex, baseline left DG volume, and diagnosis (F (1,13) = 4.8, P = 0.047, partial η^2^ = 0.27).

**Discussion:**

The findings of this study suggest that rs1079610 in OPN4 might be a potential SNP that affects volumetric change in the left DG following light therapy in mood disorders.

## 1 Introduction

Bright light therapy (BLT) is an established standard treatment for seasonal affective disorder (SAD) and has been explored for its efficacy in other mood disorders, including major depressive disorder (MDD) and bipolar disorder (BP) (1). Several mechanisms have been proposed to explain the therapeutic effects of BLT, such as modulation of circadian rhythms via the suprachiasmatic nucleus, regulation of melatonin secretion, circadian rhythm advancement, and interactions with serotonin pathways (1). However, the precise mechanism underlying BLT remains unclear.

Adult neurogenesis predominantly occurs in the hippocampal dentate gyrus (DG) (2). Antidepressant treatment has been shown to restore hippocampal neurogenesis and normalize depressive behaviors in animal models, suggesting that deficits in hippocampal neurogenesis may be a potential target for depression (3). A study reported that 4-week bright light (BL) induced neurogenesis in the adult rat hippocampal DG using 5-bromo-2’-deoxyuridine which is the marker of cell proliferation (4). Thus, we hypothesized that BL exposure could similarly induce neurogenesis in the human hippocampus, particularly in the DG. We previously reported that four weeks of BL exposure in patients with mood disorders led to increased volume of the left DG (5). However, in our previous research, while some participants exhibited an increase in the left DG volume following BL exposure, others did not (5). These findings suggest that individual sensitivity to light may vary, potentially influencing the occurrence, and genetic predisposition may contribute to this variability. Single-nucleotide polymorphisms (SNPs) are common genetic variations associated with individual traits and predispositions. The retinal cells convert the light to electrical signals, which are transmitted along the neural pathways in the brain. Melanopsin is a blue light-sensitive opsin-type G-protein coupled receptor that is expressed in a small subset of retinal ganglion cells in the human retina, termed intrinsically photosensitive retinal ganglion cells (ipRGCs) (6). The rs1079610 polymorphism in the opsin 4 (OPN4) gene has been associated with the pupillary light reflex and may therefore influence individual sensitivity to light.

On the basis of these findings, we propose a novel hypothesis that the extent of light-induced volumetric increase in the left DG may be influenced by individual genetic variation in the rs1079610 of the OPN4 gene. This exploratory study aimed to test this hypothesis.

## 2 Methods

### 2.1 Participants

This study involved recontacting patients with mood disorders who had previously participated in our research initiated in October 2015 and completed in February 2021,^5^, ^8^ a 4-week randomized controlled trial in which participants were assigned to either a BL group (10,000 lux) or a DL group (50 lux), using the envelope method in a 1:1 ratio. The random allocation sequence was generated by HH, and HH enrolled the patients. The participants then drew envelopes and were assigned to the BL or DL group. The outcome of the randomization was not concealed. The daily light exposure protocol was administered for 30 min between 7:00 and 7:30 a.m. every morning for 4 weeks. At the start of the study, participants were instructed to maintain their usual lifestyle throughout the trial. Patients with mood disorders continued their current medications and dosages unchanged during the study period. For the present study, participants contacted were provided with detailed information regarding the current study, and informed consent was obtained from those who agreed to participate. For these individuals, new blood samples were collected for genetic analysis, and additional consent was obtained to utilize their previously acquired brain imaging data. Eligible participants were recruited from the patients visiting Oita University Hospital. The inclusion criteria included a diagnosis of MDD or BP according to the Diagnostic and Statistical Manual of Mental Disorders (DSM)-IV-TR or DSM-5 criteria, age 20 years or older, right-handedness, and stable psychotropic medication use for at least one month before enrollment. All participants were screened using the Mini-International Neuropsychiatric Interview to identify coexisting psychiatric disorders. Exclusion criteria were serious physical illnesses, cognitive impairment affecting decision-making abilities, inability to provide consent, pregnancy, or lactation. Written informed consent was obtained from all participants. Participation was withdrawn if consent was revoked. A more detailed description of the methodology is presented in the previous study (5, 8). The study adhered to ethical standards and relevant national and institutional guidelines for human experimentation, in accordance with the principles outlined in the 1975 Declaration of Helsinki (revised in 2008). The study protocol was approved by the Institutional Review Board of the Oita University Faculty of Medicine (2953) and registered with UMIN (registration number UMIN000056064).

### 2.2 Image acquisition, and processing

All participants underwent magnetic resonance imaging (MRI) and ^18^F-FDG positron emission tomography (PET) at baseline and after 4 weeks of light exposure. MRI data were collected using a 3T MRI scanner (MAGNETOM Verio, Siemens or MAGNETOM Skyra Fit, Siemens) equipped with a 32-channel array head coil and a gradient strength of 45 mT/m. T1-weighted structural images were acquired using a 3D magnetization-prepared rapid gradient-echo sequence in the sagittal plane. MRI data from 9 patients were acquired using a Siemens MAGNETOM Verio scanner between October 2015 and September 2018, whereas MRI data from 15 patients were acquired using a Siemens MAGNETOM Skyra fit scanner between October 2018 and February 2021. All MRI preprocessing and segmentation procedures in the present analysis were performed using the FreeSurfer algorithm. MRI data were processed using the longitudinal stream in FreeSurfer 7.1.1 to obtain reliable volume estimates (9). Longitudinal hippocampal subfield segmentation was performed via the FreeSurfer 7.1.1 hippocampal subfields module to evaluate the volumes of the bilateral granule cell and molecular layers of the DG-head and DG-body (10, 11). This approach has been shown to yield significantly lower volume differences and significantly higher Dice overlaps than cross-sectional segmentation methods in test–retest reliability experiments (average across subregions: 4.5% vs. 6.5%; Dice overlap: 81.8% vs. 75.4%) (11). The volume of the DG was computed by summing the volumes of the DG-head and DG-body. A representative example of the DG identification based on FreeSurfer segmentation is shown in **Supplementary Figure 1**. For ^18^F-FDG PET, participants fasted for at least 5 hours before receiving an intravenous injection of 3.0 MBq/kg of ^18^F-FDG. After resting for 40 minutes in a silent room, images were acquired over a 20-minute period using a Siemens Biograph PET/CT scanner. We used 0–20 minutes of summed data, and bilateral hippocampal standardized uptake value ratio (SUVr) values were calculated using PET Surfer analysis in FreeSurfer 7.1.1 (12, 13).

### 2.3 Genotyping

Genomic DNA was extracted from whole blood samples using standard procedures. Based on the primary hypothesis of this study, rs1079610 in the OPN4 gene was genotyped. In addition, rs2675703 in OPN4 was analyzed as an exploratory SNP, as this variant has previously been reported to be associated with seasonal affective disorder (SAD), a condition in which light is thought to play an important role in the pathophysiology and for which BLT has been shown to be effective (14). The major and minor alleles for each SNP were verified using the National Center for Biotechnology Information (NCBI) SNP database (https://www.ncbi.nlm.nih.gov/SNP).

Genotype analysis was performed by the Foundation for Promotion of Material Science and Technology of Japan, and genotype analysts were blinded to treatment allocation and clinical information. SNP genotyping was performed on a MassARRAY Analyzer 4 CPM 384 (Agena Bioscience, San Diego, CA) using the Complete iPLEX Pro Genotyping Reagent Set. Data management and analyses were conducted by MassArrayTyper 4.0 Software (Agena Bioscience). Polymerase chain reaction was performed according to the instructions of the manufacturers. More detailed information about primers and polymerase chain reaction conditions for genotyping is available upon request. Genotyping quality was assessed by evaluating genotype call rates, minor allele frequencies (MAF), and Hardy–Weinberg equilibrium (HWE). HWE was evaluated using a chi-square goodness-of-fit test. All study participants were Japanese individuals recruited from a single-center cohort. Because the study population was ethnically homogeneous, substantial population stratification was considered unlikely. The study followed the Strengthening the Reporting of Genetic Association study (STREGA) statement which is an extension of the Strengthening the Reporting of Observational Studies in Epidemiology statement for reporting genetic association studies (15).

### 2.4 Statistical analysis

The demographic characteristics of the BL and DL groups were compared using two-sample t-tests and chi-square tests. To evaluate changes in brain imaging, percentage changes (%Δ) were used. The percentage change in the volume of the left DG was calculated as ([post volume of the left DG − baseline volume of the left DG]/baseline volume of the left DG) × 100. The percentage change in volume was denoted as %ΔV, and the percentage change in the standardized uptake value ratio (SUVr) was denoted as %ΔU. Given the small sample size and the low frequency of homozygous minor allele genotypes, associations between SNPs and imaging outcomes were assessed using dominant genetic models.

The primary outcome was the main effect of the rs1079610 genotype for %ΔV of the left DG. Analysis of covariance (ANCOVA) was conducted using a general linear model. %ΔV of the left DG was included as the dependent variable, whereas treatment group (BL versus DL), rs1079610 genotype (TT versus C carriers), and their interaction were included as fixed factors (Model 1). Sensitivity analyses were subsequently conducted with sequential adjustment for (Model 2) age and sex, (Model 3) age, sex, and baseline left DG volume, and (Model 4) age, sex, baseline left DG volume, and diagnosis (MDD versus BP). Because the association between the rs1079610 genotype and %ΔV of the left DG represented the primary hypothesis-driven analysis, statistical significance was assessed using a two-sided α level of 0.05 without adjustment for multiple comparisons.

Exploratory analyses for hypothesis-generating examined associations of rs1079610 with %ΔV of the right DG and %ΔU of the bilateral hippocampus, as well as associations of rs2675703 with %ΔV of the bilateral DG and %ΔU of the bilateral hippocampus. Given the exploratory nature of these analyses, the resulting p-values should be interpreted as descriptive and hypothesis-generating rather than confirmatory. All statistical analyses were performed using SPSS Statistics version 28.0 (IBM Corp., Armonk, NY, USA).

## 3 Results

We recontacted 24 patients with mood disorders who had previously participated in our study via telephone; however, three were unreachable. Informed consent was obtained from the remaining 21 patients, resulting in a final sample size of 21 (BL group, n = 11; DL group, n = 10) (**Table 1**). Genotype calls were successfully obtained for all participants for both SNPs (call rate = 100%). The MAF were 0.262 for rs1079610 and 0.167 for rs2675703. Both SNPs were in HWE (rs1079610, P = 0.81; rs2675703, P = 0.91). Demographic characteristics, MRI scanner, psychiatric comorbidities, psychotropic medication use, DG volume, hippocampal SUVr, and genotypes of SNPs were comparable between groups (**Table 2**).

**Table 1:** The demographic characteristics of all patients with mood disorder.

Demographic variables	Patients with mood disorder (n=21)
Age mean (s.d.)	38.3 (10.7)
Sex (Male, Female)	10, 11
Diagnosis	MDD: 16, BP: 5
MRI (Verio scanner, Skyra fit scanner)	6, 15
Baseline DG Volume mm^3^ mean (s.d.)	
Left DG	283.7 (32.4)
Right DG	292.5 (29.5)
Post DG Volume mm^3^ mean (s.d.)	
Left DG	285.7 (31.6)
Right DG	295.2 (28.5)
Percentage changes of DG Volume mean (s.d.)	
%ΔV left DG	0.77 (1.95)
%ΔV right DG	1.0 (2.42)
Baseline PET SUVr mean (s.d.)	
Left hippocampus	1.29 (0.08)
Right hippocampus	1.32 (0.07)
Post PET SUVr mean (s.d.)	
Left hippocampus	1.31 (0.08)
Right hippocampus	1.34 (0.07)
Percentage changes of hippocampus SUVr mean (s.d.)	
%ΔU left hippocampus	1.22 (3.15)
%ΔU right hippocampus	1.26 (2.83)
Genotype SNP_Gene	
rs1079610_OPN4	TT:12, CT:7, CC:2
rs2675703_OPN4	CC:13, CT:6, TT:2

Bipolar disorder, BP, Dentate gyrus, DG, Magnetic resonance imaging, MRI, Major Depressive Disorder, MDD, Standard deviation, s.d.

**Table 2:** The demographic characteristics of BL group and DL group.

Demographic variables	Patients with mood disorder (n=24)		p value
	BL group (n=11)	DL group (n=10)	
Age mean (s.d.)	35.9 (10.9)	41.0 (10.3)	0.29
Sex (Male, Female)	6, 5	5, 5	0.59
Diagnosis	MDD: 7, BP: 4	MDD: 9, BP: 1	0.19
MRI (Verio scanner, Skyra fit scanner)	3, 8	3, 7	0.63
Comorbidity	Panic: 2, SAD:1	Panic: 4, SAD: 2, GAD: 1	
Psychotropics	Antidepressant:10 (Esci:2, Mirt:2, Sert:1, Paro:2, Dulo:1, Venl:1, Traz:1), Mood stabilizer:5 (LTG:3, Li:2), Antipsychotics:6 (QUE:3, ARI:1, LEV:2), Hypnotics:8	Antidepressant:14 (Esci:2, Dulo:3, Mirt:6, Sert:1, Paro:1, Dosu:1), Mood stabilizer:6 (LTG:3, Li:2, VPA:1), Antipsychotics:5 (QUE:1, ARI:2, OLA:1, SUL:1), Hypnotics:8	
Baseline DG Volume mm^3^ mean (s.d.)			
Left DG	283.2 (34.1)	284.2 (32.2)	0.47
Right DG	295.5 (32.7)	289.2 (26.9)	0.32
Post DG Volume mm^3^ mean (s.d.)			
Left DG	287.5 (34.2)	283.7 (30.2)	0.4
Right DG	297.8 (30.5)	292.3 (27.4)	0.33
Percentage changes of DG Volume mean (s.d.)			
%ΔV left DG	1.55 (1.33)	-0.09 (2.22)	0.051
%ΔV right DG	0.91 (2.49)	1.11 (2.46)	0.86
Baseline PET SUVr mean (s.d.)			
Left hippocampus	1.28 (0.1)	1.29 (0.05)	0.9
Right hippocampus	1.32 (0.08)	1.32 (0.06)	0.96
Post PET SUVr mean (s.d.)			
Left hippocampus	1.31 (0.11)	1.3 (0.04)	0.69
Right hippocampus	1.34 (0.09)	1.33 (0.05)	0.59
Percentage changes of hippocampus SUVr mean (s.d.)			
%ΔU left hippocampus	1.69 (3.81)	0.71 (2.31)	0.49
%ΔU right hippocampus	1.86 (3.31)	0.6 (2.17)	0.32
Genotype SNP_Gene			
rs1079610_OPN4	TT:6, CT:3, CC:2	TT:6, CT:4, CC:0	0.32
rs2675703_OPN4	CC:8, CT:2, TT:1	CC:7, CT:3, TT:0	0.55

Aripiprazole, ARI, Bipolar disorder, BP, Bright light exposure group, BL group, Dentate gyrus, DG, Dim light exposure group, DL group, Dosulepin, Dosu, Duloxetine, Dulo, Escitalopram, Esci, Generalized anxiety disorder, GAD, Magnetic resonance imaging, MRI, Major Depressive Disorder, MDD, Mirtazapine, Mirt, Levomepromazine, LEV, Lithium, Li, Lamotrigine, LTG, Olanzapine, OLA, Panic disorder, Panic, Paroxetine, Paro, Quetiapine, QUE, Sertraline, Sert, Sulpiride, SUL, Social anxiety disorder, SAD, Standard deviation, s.d., Trazodone, Traz, Valproate, VPA, Venlafaxine, Venl.

**Figure 1:**
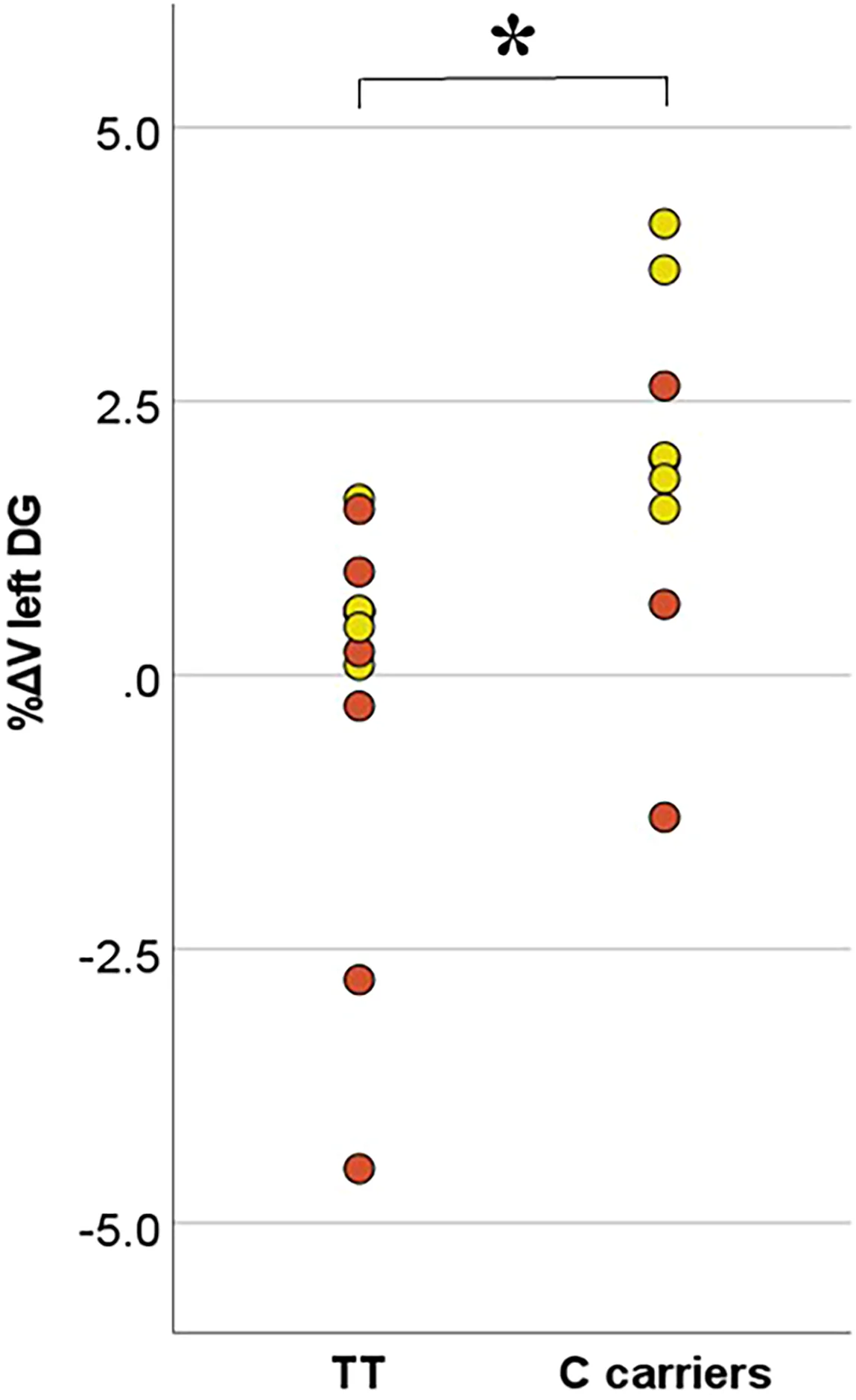
Differences in changes in left hippocampal dentate gyrus volume according to rs1079610 variants in OPN4 under dominant models. Yellow circle represents the bright group, while red circle represents the dim light group. Analysis of covariance was conducted using a general linear model. The significant main effect was shown in rs1079610 genotype (C allele carriers versus TT) under the dominant model (F(1,17) = 7.16, P = 0.016, partial η^2^ = 0.3). *Indicates that the p values < 0.05.

Under the dominant genetic model, the association between the rs1079610 genotype and %ΔV in left DG was examined using ANCOVA (**Table 3**; **Figure 1**). In the primary model (Model 1), a significant main effect of rs1079610 genotype (F (1,17) = 7.16, P = 0.016, partial η^2^ = 0.3) and treatment group (F (1,17) = 4.81, P = 0.043, partial η^2^ = 0.22) was observed, whereas the interaction between rs1079610 genotype and treatment group was not significant. Sensitivity analyses were performed with sequential adjustment for potential confounders. The association between rs1079610 genotype and %ΔV of the left DG remained statistically significant after adjustment for age and sex (Model 2: F(1,15) = 7.0, P = 0.018, partial η^2^ = 0.32), age, sex, and baseline left DG volume (Model 3: F(1,14) = 5.57, P = 0.033, partial η^2^ = 0.29), and age, sex, baseline left DG volume, and diagnosis (Model 4: F(1,13) = 4.8, P = 0.047, partial η^2^ = 0.27). The significant association between the rs1079610 genotype and %ΔV of the left DG remained across all models. No significant effects of treatment group, genotype-by-treatment interaction, age, sex, baseline left DG volume, or diagnosis were observed in any of the models (**Table 3**).

**Table 3:** Association between rs1079610 genotype and percentage change in left dentate gyrus volume under dominant models.

Effect	Model 1 F (P, partial η^2^)	Model 2 F (P, partial η^2^)	Model 3 F (P, partial η^2^)	Model 4 F (P, partial η^2^)
rs1079610 genotype	7.16 (0.016*, 0.3)	7.0 (0.018*, 0.32)	5.57 (0.033*, 0.285)	4.8 (0.047*, 0.27)
Treatment group	4.81 (0.043*, 0.22)	3.97 (0.065, 0.21)	3.94 (0.067, 0.22)	2.92 (0.11, 0.18)
Genotype × Treatment group	0.02 (0.9, 0.001)	0.06 (0.82, 0.004)	0.11 (0.75, 0.008)	0.14 (0.72, 0.01)
Age	–	0.008 (0.93, 0.001)	0.006 (0.94, 0.000)	0.015 (0.91, 0.001)
Sex	–	0.58 (0.46, 0.04)	0.34 (0.57, 0.02)	0.38 (0.55, 0.03)
Baseline left DG volume	–	–	0.59 (0.45, 0.04)	0.55 (0.47, 0.04)
Diagnosis	–	–	–	0.14 (0.72, 0.01)

Bold values and * indicates that the p values < 0.05.

Results of the exploratory analyses are presented in **Supplementary Tables 1**, **2**. Overall, no consistent associations were observed between OPN4 variants (rs1079610 and rs2675703) and volumetric or metabolic changes in the examined regions.

## 4 Discussion

The current study revealed that the significant association between rs1079610 genotype (C allele carriers versus TT) and left DG volumetric change following light therapy in mood disorders. Furthermore, this main effect remained significant after adjustment for age, sex, baseline left DG volume, and diagnosis. However, contrary to our initial expectation, no significant genotype-by-treatment group was detected, indicating that the association between the rs1079610 genotype and left DG volumetric change did not significantly differ between the bright light and dim light groups. Therefore, these results partially support our hypotheses.

Melanopsin is a photopigment expressed on the cellular membranes of ipRGCs. IpRGCS transmit light energy into electrical signals to limbic structures, such as the hippocampus, suggesting that light may directly influence mood regulation (16). These melanopsin‐expressing ipRGCs mediate non–image‐forming visual functions, including entraining of circadian rhythm, pupillary light responses, endocrine regulation, and sleep–wake cycles.^6^, ^17^ Like other genes, naturally occurring mutations and sequence changes are known to exist within the human melanopsin gene. In rs1079610, under high illuminance conditions, C allele carriers exhibited significantly smaller pupil sizes and a greater percentage of pupil constriction compared to those with the TT genotype (7). Another study reported that the pupils of the C allele carriers were significantly smaller than those of the TT genotype under a blue light condition (18). In addition, relative pupil constrictions of the C allele carriers were greater than those of the TT genotype under both blue and green conditions with high intensities (18).These findings suggest that C allele carriers of rs1079610 may be more sensitive to high-intensity light than individuals with the TT genotype. Recently, a study investigated the associations between melanopsin genetic variants, sleep, and markers of brain health in cognitively unimpaired older adults, revealing that the interactions of the rs1079610 with sleep disturbance, daytime dysfunction, and sleep duration were significantly associated with language (19). TT homozygotes had decreasing language scores with worsening sleep measures for sleep disturbance, and daytime dysfunction, whereas these association were not significant for people with the C allele carriers. Therefore rs1079610, associated with cognition, might be interacting with sleep traits (19). Although the functional consequences of rs1079610 remain unclear, accumulating evidence suggests that this variant may influence individual sensitivity to light. In this context, previous findings may be consistent with our observation that C allele carriers of rs1079610 exhibited greater light-induced volumetric increases in the left DG following four weeks of light exposure. Rather, the findings suggest that rs1079610 may be associated with individual differences in the left DG structural plasticity irrespective of treatment condition. Because the present study included a small sample size, the statistical power to detect interaction effects was limited.

We also investigated rs2675703, another OPN4 variant which is associated with SAD (14), in an exploratory analysis; however, no consistent associations were observed between rs2675703 and volumetric or metabolic changes in the examined regions. Therefore, from a hypothesis-generating perspective, rs1079610 may have a greater influence on structural plasticity in the left DG than rs2675703.

Several limitations of the present study should be acknowledged. First, the exploratory nature of the analyses increases the possibility of type I errors. Second, although participants were randomly allocated using sealed envelopes, allocation concealment was not implemented because the investigator who generated the allocation sequence also enrolled participants and was aware of treatment assignment. Therefore, the possibility of selection bias cannot be completely excluded. In addition, MRI image analyses were not performed under blinded conditions with respect to treatment allocation and clinical information. However, volumetric and SUVR measurements were generated automatically using standardized FreeSurfer-based image processing, minimizing operator-dependent assessment. Third, the heterogeneity of medication use among participants may have influenced the observed structural and metabolic changes. Forth, the inclusion of both MDD and BP patients may have introduced diagnostic heterogeneity. In addition, variability across MRI scanners and acquisition conditions may have contributed to measurement noise. Fifth, the relatively small sample size may affect the generalizability of the findings. Furthermore, we could not conduct sensitivity analyses using regression models adjusted for potential confounders such as MRI scanner type, medication class, season, sleep-wake rhythm, baseline symptom severity, and comorbid anxiety disorders, due to the small sample size.

In conclusion, the findings of this study suggest that rs1079610 in OPN4 might be a potential SNP that affects volumetric change in the left DG following light therapy in mood disorders. Further studies involving larger sample sizes and molecular assays are needed to confirm and extend these findings.

## Data Availability

The raw data supporting the conclusions of this article will be made available by the authors, without undue reservation.
