# Supplementary material for: Melanopsin gene polymorphism and volumetric change in the left hippocampal dentate gyrus following light therapy in mood disorders: An exploratory study
Source: Front Psychiatry. 2026 Aug 13;17:1876421. doi: 10.3389/fpsyt.2026.1876421 (PMC13518336; doi:10.3389/fpsyt.2026.1876421)
Supplement: Supplementary file 1 [file Table1.docx]

Supplementary Material

# 1 Supplementary Tables

**Table S1.** Association between the rs1079610 genotype and percentage change in right dentate gyrus volume and percentage change in bilateral hippocampus metabolism under dominant models.

| Effect | Model 1 F (P, Partial η²) | Model 2 F (P, Partial η²) | Model 3 F (P, Partial η²) | Model 4 F (P, Partial η²) |
| --- | --- | --- | --- | --- |
| **%ΔV right DG** |  |  |  |  |
| rs1079610 genotype | 0.23 (0.64, 0.01) | 0.29 (0.6, 0.02) | 0.02 (0.89, 0.001) | 0.03 (0.87, 0.002) |
| Treatment group | 0.02 (0.9, 0.001) | 0.007 (0.93, <0.0001) | 0.12 (0.73, 0.009) | 0.15 (0.7, 0.01) |
| Genotype × Treatment group | 6.15 (0.02, 0.27) | 5.63 (0.03, 0.27) | 4.12 (0.06, 0.23) | 3.55 (0.08, 0.22) |
| Age | – | 0.006 (0.94, <0.0001) | 0.18 (0.68, 0.01) | 0.19 (0.67, 0.02) |
| Sex | – | 0.41 (0.53, 0.03) | 0.29 (0.6, 0.02) | 0.22 (0.65, 0.02) |
| Baseline right DG volume | – | – | 1.55 (0.23, 0.1) | 1.48 (0.25, 0.1) |
| Diagnosis | – | – | – | 0.05 (0.83, 0.004) |
|  |  |  |  |  |
| **%ΔU left hippocampus** |  |  |  |  |
| rs1079610 genotype | 1.26 (0.28, 0.07) | 0.89 (0.36, 0.06) | 0.47 (0.51, 0.03) | 0.46 (0.51, 0.03) |
| Treatment group | 0.66 (0.43, 0.04) | 0.7(0.42, 0.05) | 0.62 (0.44, 0.04) | 0.6 (0.45, 0.04) |
| Genotype × Treatment group | 1.35 (0.26, 0.07) | 0.83 (0.38, 0.05) | 0.62 (0.44, 0.04) | 0.54 (0.48, 0.04) |
| Age | – | 0.03 (0.88, 0.002) | 0.008 (0.93, 0.001) | 0.01 (0.91, 0.001) |
| Sex | – | 1.06 (0.32, 0.07) | 1.15 (0.3, 0.08) | 1.09 (0.32, 0.08) |
| Baseline left hippocampus PET SUVr | – | – | 0.15 (0.7, 0.01) | 0.12 (0.74, 0.009) |
| Diagnosis | – | – | – | 0.03 (0.86, 0.002) |
|  |  |  |  |  |
| **%ΔU right hippocampus** |  |  |  |  |
| rs1079610 genotype | 0.18 (0.68, 0.01) | 0.06 (0.82, 0.004) | 0.001 (0.98, <0.0001) | 0.02 (0.9, 0.001) |
| Treatment group | 1.69 (0.21, 0.09) | 2.08 (0.17, 0.12) | 1.82 (0.2, 0.12) | 1.97 (0.18, 0.13) |
| Genotype × Treatment group | 3.42 (0.08, 0.17) | 2.15 (0.16, 0.13) | 1.39 (0.26, 0.09) | 1.17 (0.3, 0.08) |
| Age | – | 0.34 (0.57, 0.02) | 0.18 (0.68, 0.01) | 0.23 (0.64, 0.02) |
| Sex | – | 1.94 (0.18, 0.11) | 2.11 (0.17, 0.13) | 2.16 (0.17, 0.14) |
| Baseline right hippocampus PET SUVr | – | – | 0.27 (0.61, 0.02) | 0.2 (0.67, 0.02) |
| Diagnosis | – | – | – | 0.25 (0.63, 0.02) |

Abbreviation; Dentate gyrus: DG.

**Table S2.** Association between the rs2675703 genotype and percentage change in bilateral dentate gyrus volume and percentage change in bilateral hippocampus metabolism under dominant models.

| Effect | Model 1 F (P, Partial η²) | Model 2 F (P, Partial η²) | Model 3 F (P, Partial η²) | Model 4 F (P, Partial η²) |
| --- | --- | --- | --- | --- |
| **%ΔV left DG** |  |  |  |  |
| rs2675703 genotype | 3.7 (0.07, 0.18) | 3.64 (0.08, 0.2) | 2.75 (0.12, 0.16) | 2.25 (0.16, 0.15) |
| Treatment group | 3.13 (0.1, 0.16) | 2.83 (0.11, 0.16) | 2.88 (0.11, 0.17) | 2.5 (0.14, 0.16) |
| Genotype × Treatment group | 0.37 (0.55, 0.02) | 0.45 (0.51, 0.03) | 0.27 (0.62, 0.02) | 0.18 (0.68, 0.01) |
| Age | – | 0.25 (0.63, 0.02) | 0.22 (0.64, 0.02) | 0.18 (0.68, 0.01) |
| Sex | – | 0.18 (0.68, 0.01) | 0.2 (0.66, 0.01) | 0.13 (0.73, 0.01) |
| Baseline left DG volume | – | – | 0.54 (0.47, 0.04) | 0.51 (0.49, 0.04) |
| Diagnosis | – | – | – | 0.01 (0.91, 0.001) |
|  |  |  |  |  |
| **%ΔV right DG** |  |  |  |  |
| rs2675703 genotype | 2.04 (0.17, 0.11) | 2.28 (0.15, 0.13) | 0.67 (0.43, 0.05) | 0.96 (0.35, 0.07) |
| Treatment group | 0.02 (0.88, 0.001) | 0.002 (0.97, <0.0001) | 0.02 (0.9, 0.001) | 0.1 (0.76, 0.007) |
| Genotype × Treatment group | 0.003 (0.96, <0.0001) | 0.02 (0.9, 0.001) | 0.13 (0.72, 0.009) | 0.44 (0.52, 0.03) |
| Age | – | 0.69 (0.42, 0.04) | 1.28 (0.28, 0.08) | 1.62 (0.23, 0.11) |
| Sex | – | 0.11 (0.75, 0.007) | 0.09 (0.78, 0.006) | 0.36 (0.56, 0.03) |
| Baseline right DG volume | – | – | 1.43 (0.25, 0.09) | 1.6 (0.23, 0.11) |
| Diagnosis | – | – | – | 0.78 (0.39, 0.06) |
|  |  |  |  |  |
| **%ΔU left hippocampus** |  |  |  |  |
| rs2675703 genotype | 0.65 (0.43, 0.04) | 1.73 (0.21, 0.1) | 1.14 (0.3, 0.08) | 1.47 (0.25, 0.1) |
| Treatment group | 0.46 (0.51, 0.03) | 0.33 (0.58, 0.02) | 0.23 (0.64, 0.02) | 0.38 (0.55, 0.03) |
| Genotype × Treatment group | 0.01 (0.92, 0.001) | 0.51 (0.49, 0.03) | 0.55 (0.47, 0.04) | 0.86 (0.37, 0.06) |
| Age | – | 0.55 (0.47, 0.04) | 0.29 (0.6, 0.02) | 0.43 (0.52, 0.03) |
| Sex | – | 3.25 (0.09, 0.18) | 3.52 (0.08, 0.2) | 3.88 (0.07, 0.23) |
| Baseline left hippocampus PET SUVr | – | – | 0.42 (0.53, 0.03) | 0.27 (0.61, 0.02) |
| Diagnosis | – | – | – | 0.49 (0.5, 0.04) |
|  |  |  |  |  |
| **%ΔU right hippocampus** |  |  |  |  |
| rs2675703 genotype | 0.24 (0.63, 0.01) | 1.42 (0.25, 0.09) | 0.65 (0.43, 0.04) | 1.5 (0.24, 0.1) |
| Treatment group | 0.81 (0.38, 0.05) | 0.84 (0.38, 0.05) | 0.61 (0.45, 0.04) | 1.19 (0.3, 0.08) |
| Genotype × Treatment group | <0.0001 (0.99, <0.0001) | 0.9 (0.36, 0.06) | 0.97 (0.34, 0.07) | 2.04 (0.18, 0.14) |
| Age | – | 1.58 (0.23, 0.1) | 0.71 (0.41, 0.05) | 1.34 (0.27, 0.09) |
| Sex | – | 4.83 (0.04, 0.24) | 5.01 (0.04, 0.26) | 6.96 (0.02, 0.35) |
| Baseline right hippocampus PET SUVr | – | – | 0.5 (0.49, 0.03) | 0.22 (0.64, 0.02) |
| Diagnosis | – | – | – | 1.85 (0.2, 0.12) |

Abbreviation; Dentate gyrus: DG.

# 2 Supplementary Figure

# Figure S1 A representative example of the dentate gyrus identification based on FreeSurfer segmentation.

A representative example of the dentate gyrus (DG) identification based on FreeSurfer segmentation in the present study. The light blue region represents the dentate gyrus.
